# The calcium–phosphate balance, modulation of thyroid autoimmune processes and other adverse effects connected with thyroid arterial embolization

**DOI:** 10.1007/s12020-013-0072-2

**Published:** 2013-10-22

**Authors:** Grzegorz Kaminski, Andrzej Jaroszuk, Ariadna Zybek, Krzysztof Brzozowski, Piotr Piasecki, Piotr Ziecina, Marek Ruchala

**Affiliations:** 1 Department of Endocrinology and Isotope Therapy, Military Institute of Medicine, Szaserów Street 128, 04-141 Warsaw, Poland; 2 Department of Endocrinology, Metabolism and Internal Medicine, Poznan University of Medical Sciences, Przybyszewskiego Street 49, 60-355 Poznan, Poland; 3 Department of Interventional Radiology, Military Institute of Medicine, Szaserów Street 128, 04-141 Warsaw, Poland

**Keywords:** Thyroid embolization, Thyroid diseases, Calcium–phosphate balance, Thyroid autoimmunity, Side effects

## Abstract

In search of new treatment options for thyroid diseases, when conventional procedures are ineffective, contraindicated or associated with serious side effects, safety of thyroid arteries embolization in the treatment of particular thyroid diseases was evaluated. The study included eight subjects with retrosternal toxic goiter, six patients affected by Graves’ disease, five cases of retrosternal non-toxic goiter, two subjects with post-amiodarone hyperthyroidism, and one patient with severe thyroid-related orbitopathy, who underwent selective embolization of thyroid arteries. The study assessed and compared calcium–phosphate balance, modulation of thyroid autoimmunity and the presence of different side effects in patients who underwent the procedure. In addition, the serum concentrations of thyroid hormones, anti-thyroid autoantibodies and thyroid volume have been measured. Five of all enrolled subjects (22.7 %) experienced transient, not clinically relevant hypocalcaemia with no need for calcium supplementation. There were no significant changes in serum calcium levels in patients after embolization of both inferior thyroid arteries. The transient side effects associated with the treatment were neck pain and a slight increase in body temperature. Noted high concentration of free thyroid hormones immediately after the procedure was not accompanied by worsening of symptoms or signs of thyrotoxicosis. In patients with Graves’ disease, a significant decrease in thyrotropin receptor antibodies level was observed. Thyroid arterial embolization does not disturb permanently calcium–phosphate balance, modulates positively thyroid autoimmune processes and is associated with no serious post-procedure side effects. Hence, it may be considered as a safe and effective treatment modality for selected thyroid disorders.

## Introduction

Thyroid gland diseases remain one of the most widespread problems in the modern clinical practice. It has been estimated that almost 9 % of women and 2 % of men are affected by a thyroid disorder [1]. Despite the existence of effective therapies for thyroid diseases, still some clinical cases require individual and alternative treatment modalities that would satisfy both patient and physician [2]. Standard therapeutic methods of hyperthyroidism include anti-thyroid drug therapy, radioiodine treatment with or without human TSH stimulation, and surgical procedures. Medical therapy may be connected with serious allergic reactions, agranulocytosis, thrombocytopenia, liver damage and many other adverse effects [3–7]. Anti-thyroid drugs intake during pregnancy sometimes results in congenital malformations and hypothyroidism of the fetus [3]. Effectiveness of the radioiodine intake may be easily reduced by earlier use of amiodarone, contrast agents, fast iodine metabolism or low iodine uptake in the thyroid [8–13]. In addition, there is a risk of orbitopathy development or deterioration during the radioiodine treatment [14, 15]. The side effects associated with surgical procedures include post-operative hypoparathyroidism, recurrent laryngeal nerve damage and hemorrhage into the post-operative space [16–21].

The arterial embolization has been already widely used by interventional radiologists to treat vascular changes [22] as well as in oncology as a help in bleeding control and pain reduction or in various neoplasms management combined with locally administered chemotherapy [23, 24]. The efficacy of this technique in thyroid diseases was firstly evaluated by Galkin et al. [25], providing the evidence of thyroid volume reduction and prevention of goiter recurrence. Available data confirm usefulness of this method in patients affected by toxic goiter, recurrent goiter, Graves’ disease (GD) or even thyroid carcinoma [2, 26–31]. The previous study conducted in our department supports these observations by showing that thyroid arterial embolization may be offered as an effective alternative for patients who will not or cannot accept standard therapy [32]. However, the influence of this procedure on calcium–phosphate balance and the autoimmune processes has not been fully evaluated to date.

The aim of our study was to assess the safety of thyroid arterial embolization in patients affected by various thyroid disorders.

## Subjects and methods

### Subjects

The studied group consisted of 22 consecutive patients (19 women and 3 men) aged from 18 to 83 years (mean 55.9 ± 15.4 years) referred to the department between 2004 and 2010, who met the inclusion criteria. The studied group included eight subjects with retrosternal toxic goiter, six patients affected by GD, five cases of retrosternal non-toxic goiter, two subjects with post-amiodarone hyperthyroidism, and one patient with severe thyroid-related orbitopathy. The enrolled group is characterized in Table 1. Thyrotoxicosis was in all affected patients caused by hyperthyroidism, and all cases of destructive thyrotoxicosis were excluded from the study.

**Table 1:** Characteristics of the patients enrolled in the study

Patient no.	Age	Diagnosis/complication	Pressure signs yes/no	Retrosternal goiter yes/no	Recurrence yes/no	Thyroid volume (ml)
1	77	Multinodular toxic goiter	Yes	Yes	Yes	238
2	60	GD/unsuccessful ^131^I treatment	No	No	No	87
3	54	Post-amiodarone HT	No	No	No	39
4	73	Multinodular toxic goiter	Yes	Yes	No	269
5	74	Multinodular toxic goiter	Yes	Yes	No	158
6	44	Multinodular toxic goiter	Yes	Yes	No	265
7	55	Multinodular toxic goiter	Yes	Yes	No	66
8	72	Post-amiodarone HT	No	No	No	28
9	52	GD/resistant orbitopathy	No	No	No	2,12
10	52	GD/unsuccessful ^131^I treatment	No	No	No	58
11	66	Multinodular toxic goiter	Yes	Yes	No	315
12	83	Multinodular toxic goiter	Yes	Yes	No	92
13	31	GD/anti-thyroid drugs intolerance	No	No	No	110
14	45	Multinodular toxic goiter	Yes	No	No	188
15	47	GD/unsuccessful ^131^I treatment	No	No	No	87
16	18	GD/giant toxic goiter	Yes	Yes	No	211
17	51	GD/giant toxic goiter	Yes	Yes	No	263
18	48	Multinodular goiter	Yes	Yes	Yes	91
19	52	Multinodular goiter	Yes	No	No	166
20	66	Multinodular goiter	Yes	No	No	83
21	47	Multinodular toxic goiter	Yes	Yes	No	185
22	64	Multinodular goiter	Yes	No	Yes	63

*GD* Graves’ disease, *HT* hyperthyroidism

Inclusion criteria were age over 18 years old, disqualification from surgical, radioiodine or medical therapy or lack of patient’s consent to standard methods of treatment. Studied subjects were disqualified from surgical treatment because of old age, many accompanying cardiovascular and pulmonological entities or a huge, retrosternal goiter. In some cases of GD, no improvement occurred, despite a few attempts to radioiodine therapy. The post-amiodarone hyperthyroidism and multinodular goiter with only few autonomous regions are radioiodine resistant, due to low or limited thyroid parenchymal iodine uptake. Patients with anti-thyroid drug resistance or a poor medical therapy tolerance were also included into the study. One subject with active form of orbitopathy already treated with radioiodine and high doses of steroids took part in the study.

Diagnoses were based on medical histories, performed physical examinations, laboratory tests (thyroid hormones and anti-thyroid autoantibodies levels) and imaging techniques (ultrasound examination and thyroid scintiscan).

Before the embolization, all subjects underwent clinical examinations, blood tests and imaging studies. The serum concentrations of calcium (Ca), phosphate (P), anti-thyroid autoantibodies, including anti-thyroid peroxidase antibodies (AbTPO) and thyrotropin receptor antibodies (TRAb), thyroid stimulating hormone (TSH), free thyroxine (fT4), and free triiodothyronine (fT3) were measured. The size, volume and structure of the thyroid gland were pictured by computed tomography (CT) and ultrasonography (US). US-guided fine-needle aspiration biopsy of detected lesions was performed, if indicated.

The local bioethical committee approved the study, and all the participants gave written informed consent to participate.

### Arterial embolization

**Fig. 1:**
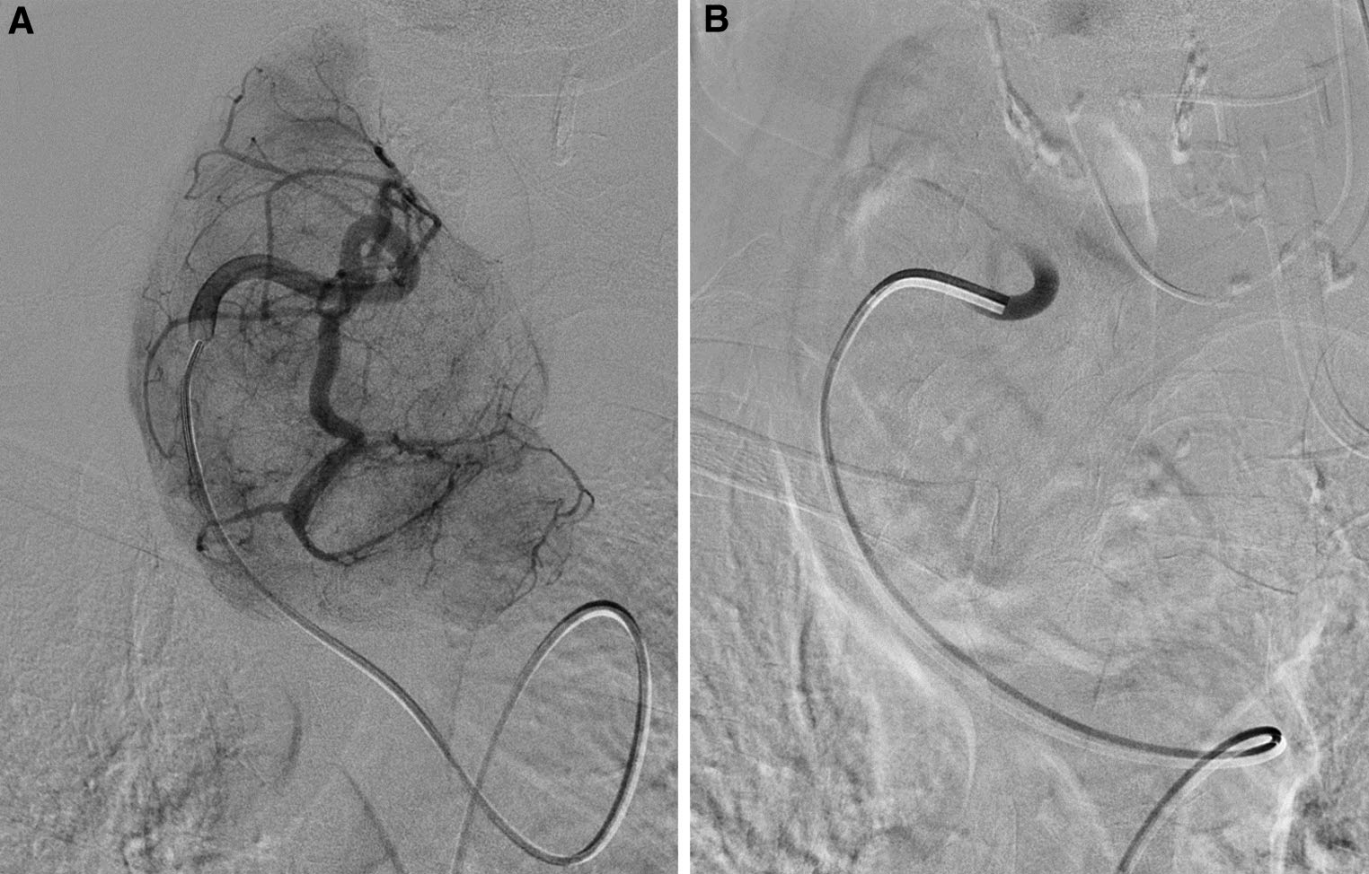
Thyroid arterial embolization in patient affected by goiter development. **a** Selective angiography of the right inferior thyroid artery before the procedure. **b** Complete occlusion of the artery showed after the successful embolization

Selective embolization of thyroid arteries was performed using the Seldinger technique. Before the procedure 1,000 ml of isotonic saline solution and 1.0 g of methylprednisolone were infused to prevent possible adverse effects connected with contrast agents’ infusion. The puncture site was chosen by the palpation of inguinal pulsation point in a patient placed in a supine position. After performing local anesthesia with 1 % procaine, a small incision was made, through which, the angiographic catheter (4F–5F) was inserted into the left or right femoral artery. The movement of the catheter to the thyroid arteries was visualized by digital subtraction angiography. Before embolization, the selective angiography was performed to estimate the regions supplied with blood by each thyroid artery and the technical ability to occlude them. That provided important information, which helped to decide about the individual range of the procedure. The arteries to be embolized were chosen by the operator to achieve maximal reduction of the goiter size compromised with the practicability of the chosen procedure variant. To prevent hypoparathyroidism, no more than three arteries were occluded at a time. In most cases of diffuse goiter, three of main thyroid arteries were embolized; however, in patients with multinodular goiter, due to frequently impaired thyroid vascularization, sometimes only one pathological vessel needed to be occluded. Polyvinyl alcohol (PVA) or the mixture of histoacryl and lipiodol was injected directly to the blood vessels. In case of PVA, the procedure began with the infusion of smaller granules (150–200 μm) to provide an effective occlusion without the risk of penetration of PVA into the systemic circulation. Subsequently, larger granules (200–300 μm) were used to definitely block the lumen of the embolized vessel. The completeness of occlusion was checked by following angiography (Fig. 1). The procedure was performed under intravenous total anesthesia, due to a pain reaction resulting from acute ischemia of the thyroid gland. Afterwards, the catheter was removed and pressure dressing was applied. The embolization took approximately 2 h. The patients were hospitalized and monitored for the next 7 days.

### Post-procedure management

All patients received pain relieving treatment with non-steroidal anti-inflammatory drugs or opioids, depending on the intensity of symptoms. Symptomatic treatment with beta-adrenergic blocking agents was introduced to prevent the occurrence of early thyrotoxicosis symptoms. The single infusion of methylprednisolone (250–500 mg) as well as treatment with prednisone (30 mg/24 h) for the following month was administered in all cases of GD.

Early post-procedure monitoring included Ca, P, TSH, fT3, fT4 level evaluation at the 1st, 3rd and 7th day after embolization, and parathormon (PTH), if hypocalcemia had occurred. The latter follow-ups, showing distant post-procedure changes, were performed after 2 weeks, 1, 2, 3, 6, 12, and on average 20.3 months and consisted of clinical examinations, TSH, fT4, and fT3 measurements. Anti-thyroid autoantibodies serum concentrations were measured at baseline, after 3 months and at the end of the observation time. Depending on the results of the 1-month follow-up visit, the anti-thyroid drug therapy, beta-adrenergic blocking agents or thyroxin supplementation were introduced. Thyroid volume evaluation with CT was performed during 12-month follow-up visit. It has been assessed with standard formula of an ellipsoid with a correction factor 0.5 (preferred range from 0.494 to 0.554) [33]. The average time of patients’ post-procedure observation was 20.3 ± 12.5 months.

### Serum assays and thyroid imaging

Anti-thyroid autoantibodies (AbTPO and TRAb) concentrations were assessed by radioimmunological method using AbTPO and TRAb RIA kits by BRAMHS. PTH level was measured with Liaison N-TACT test (normal range 17.3–72.9 pg/ml). Assessment of TSH, fT4 and fT3 serum concentration was performed using AutoDELFIA kit with a normal range of 0.55–3.55 μIU/ml, 7.64–19.7 pmol/l, and 3.5–7.9 pmol/l, respectively. Ultrasound examination was performed using Hitachi EUB 8500 with a 7.5 MHz frequency probe and computed tomography using GE LightSpeed 16.

### Statistical analysis

Comparison of Ca and P serum concentrations in patients with two inferior thyroid arteries embolized and subjects with other variant of thyroid arterial embolization (two superior and one inferior thyroid arteries embolized, two superior thyroid arteries occluded, one superior and one inferior thyroid arteries embolized, and one pathological vessel occluded) was performed with the non-parametric Mann–Whitney test. The relevant parameters (TSH, fT4, fT3, AbTPO, TRAb serum concentration and thyroid volume) were compared with the use of a nonparametric Wilcoxon test. Statistical analysis was performed using data analyzing software system STATISTICA 7.1 (Stat Soft. Inc.).

## Results

The difference in calcium level between the group of subjects with two inferior thyroid arteries embolized and subjects with other variant of thyroid arterial embolization 24 and 72 h after the procedure was not statistically significant. Five of all enrolled subjects (22.7 %) experienced transient, not clinically relevant hypocalcaemia with no need for calcium supplementation. No significant decrease in average Ca level was observed 24 and 72 h after embolization. The phosphate level was significantly higher 24 h after the procedure in patients with two inferior thyroid arteries embolized (1.361 vs 1.187 mmol/l; *p* = 0.017); however, 72 h after embolization no such difference was noted (1.099 vs 1.106 mmol/l; *p* = 0.951). All values are presented in Table 2.

**Table 2:** The mean concentrations of calcium (Ca) and phosphate (P) at baseline, 24 and 72 h after the procedure in the group of subjects with two inferior thyroid arteries embolized (2 i. t. a.) and in subjects with other variant of thyroid arterial embolization (two superior and one inferior thyroid arteries embolized, two superior thyroid arteries occluded, one superior and one inferior thyroid arteries embolized, or one pathological vessel occluded)

Parameter	Baseline		24 h		72 h	
	2 i. t. a.	Other variant	2 i. t. a.	Other variant	2 i. t. a.	Other variant
Ca [mmol/l]	2.435	2.367	2.268	2.285	2.277	2.275
	*p* = 0.251		*p* = 0.780		*p* = 0.977	
P [mmol/l]	1.225	1.192	1.361	1.187	1.099	1.106
	*p* = 0.731		*p* = 0.017		*p* = 0.951	

The serum levels of anti-thyroid autoantibodies were significantly higher in subjects with GD (6 patients) than in group not affected by this disorder (16 patients). The average change in the serum level of AbTPO and TRAb was significantly higher in the group with GD than in the second group at 3-month follow-up and at the end of the observation time. However, in subjects with GD, only the decrease in TRAb level observed at the end of the follow-up was statistically significant (26 vs 6 U/ml; *p* = 0.043). Significant changes in TRAb concentrations in subjects not affected by GD were noted neither at 3-month follow-up, nor at the end of the observation time. There was a significant increase in mean AbTPO level 3 months after the procedure, which eventually normalized after about 20.3 months (Table 3).

**Table 3:** Results of mean anti-thyroid autoantibodies (AbTPO, TRAb) serum levels assessment, at baseline and during follow-up, in patients who underwent thyroid arterial embolization

Parameter	Patients with Graves’ disease			Patients without Graves’ disease		
	Baseline	3 months	Av. 20.3 months	Baseline	3 months	Av. 20.3 months
AbTPO [U/ml]	2,031	1,194	1,436	19.8	67.02	22.27
		*p* = 0.513	*p* = 0.108		*p* = 0.023	*p* = 0.066
TRAb [U/ml]	25.73	7.9	6.06	0.074	0.058	0.15
		*p* = 0.109	*p* = 0.043		*p* = 0.770	*p* = 0.503

Each *p* value refers to the difference between the baseline value and that obtained at a particular time point. No statistical significance was reached between the values at different time points and is, therefore, not presented

The early post-procedure side effects included: neck pain (*n* = 19; 86.4 %), increased body temperature (*n* = 12; 54.5 %) and transient, not clinically relevant hypocalcaemia (*n* = 5; 22.7 %).

The mean serum concentrations of TSH, fT4 and fT3 at baseline were 0.310 μlU/ml, 17.43 pmol/l and 8.505 pmol/l, respectively. Statistically significant decrease in TSH serum level was observed 72 h and 7 days after the procedure, while during 3-month follow-up and at the end of the post-procedure observation time TSH level was within the reference range. Statistically significant increase in fT4 level was observed 24, 72 h, 7 days and 2 weeks after embolization. Similarly, fT3 serum level significantly increased 72 h, 7 days and 2 weeks after the procedure. After 2 months, the concentration of fT4 and fT3 normalized; however, fT3 level was significantly lower at the end of the follow-up than at the baseline. All the obtained values are presented in Table 4.

**Table 4:** Mean serum concentrations of TSH [μIU/ml], fT4 (*free thyroxin*) [pmol/l] and fT3 (*free triiodothyronine*) [pmol/l] during follow-up, after thyroid arterial embolization compared with values before the procedure

Parameter	Baseline	24 h	72 h	1 week	2 weeks	1 month	2 months	3 months	Av. 20.3 months
TSH [μIU/ml]	0.310	0.284	0.034	0.016	0.221	1.229	0.406	0.759	1.308
		*p* = 0.767	*p* = 0.033	*p* = 0.008	*p* = 0.074	*p* = 0.209	*p* = 0.327	*p* = 0.027	*p* = 0.0029
fT4 [pmol/l]	17.43	23.47	48.41	44.82	39.74	20.65	18.29	18.28	13.29
		*p* = 0.004	*p* = 0.0001	*p* = 0.0002	*p* = 0.0022	*p* = 0.509	*p* = 0.433	*p* = 0.664	*p* = 0.205
fT3 [pmol/l]	8.505	7.714	17.520	19.38	14.87	8.146	7.715	8.283	5.803
		*p* = 0.125	*p* = 0.015	*p* = 0.003	*p* = 0.006	*p* = 0.108	*p* = 0.722	*p* = 0.723	*p* = 0.008

Each *p* value refers to the difference between the baseline value and that obtained at a particular time point. No statistical significance was reached between the values at different time points and is, therefore, not presented

The state of euthyroidism or hypothyroidism has been reached in 70.6 % of the studied group. However, this positive effect has been achieved in 81.8 % of patients with multinodular goiter or post-amiodarone hyperthyroidism, and only in 50 % of subjects with GD.

The thyroid volume in the studied group was significantly lower at 1-year follow-up than at baseline (139.3 vs 87.72 ml, *p* = 0.00004). The mean change in thyroid volume differs significantly, depending on a number of occluded arteries (1 artery, 62.0 ml; 2 arteries, 25.6 ml; 3 arteries, 78.8 ml; *p* = 0.0401).

## Discussion

The arterial embolization is a widely described and broadly applied diagnostic and therapeutic method of modern invasive radiology. However, only few medical centers provided data about the possible advantages of arterial embolization in endocrinology [2, 25, 26, 29–32, 34].

To the best of our knowledge, full evaluation of calcium–phosphate metabolism in subjects who underwent thyroid arterial embolization has not been performed yet. As a prevention of potential parathyroid function disruption, it has been recommended not to embolize more than three main thyroid arteries and/or two inferior thyroid arteries at a time [2]. This theory was based on a fact that parathyroid glands are supplied with blood mainly by vessels originating from inferior arteries. In the current study, half of investigated cases (*n* = 12) had two inferior arteries occluded, but no significant difference in early Ca serum concentration was noted compared to other subjects. Approximately, 22.7 % of the studied group experienced transient, not clinically relevant hypocalcaemia and no calcium supplementation was needed. Generally, the average serum calcium level in the studied group during the first few days after the procedure was within the normal range. Hence, in authors’ opinion, there is no need to avoid simultaneous embolization of both inferior thyroid arteries; however, the obliteration of all four arteries may still be connected with a risk of hypoparathyroidism.

In accordance with the study by Zhao et al. [34, 35], patients with GD who underwent thyroid arterial embolization presented significant decrease in TRAb serum titer 6 months after the procedure. Results obtained in the present research seem to be in agreement with previous findings. Moreover, the serum concentration of aTPO also showed a downward trend, although it was not statistically significant. Described effect may be possibly connected with the decrease in thyroid parenchymal volume and lack of potential target for produced autoantibodies. On the other hand, in subjects without GD, a transient significant increase in aTPO serum concentration was shown that normalized at the end of the observation time. In authors’ opinion, massive necrosis of the gland may be responsible for a self-limiting immunological response that did not persist; therefore, no constant autoimmune thyroid diseases were noted as a side effect of the performed procedure.

Xiao et al. [2] reported the normalization of the thyroid function in almost 64 % of patients affected by GD (average time of observation: 6–50 months), while Zhao et al. [34] described the same positive effect in 71 % of cases after 1 year and 59 % after 3 years of observation. In the present study, 71 % of hyperthyroid patients reached euthyroid or hypothyroid state (80 % affected by multinodular goiter and 50 % by GD). The significant decrease in TRAb level and destruction of thyroid follicular cells surely play important roles in this process. Zhao et al. [30, 34] gave evidence that thyroid embolization has a significant influence on thyroid autoimmune processes, angiogenesis and apoptosis, which all together result in the remission of the disease. It is worth mentioning that patients who did not reach the euthyroid or hypothyroid state required lower doses of anti-thyroid drugs than before the therapy. The positive outcome in this group was associated with the lowering of adverse effects risk and better doctor–patient cooperation.

The evaluation of post-procedure side effects showed that most of them might have resulted from ischemia and following necrosis of the thyroid gland. The neck pain was most commonly observed with a frequency of 85 %, usually sufficiently treated only with non-steroidal anti-inflammatory drugs. Only in two cases (9 %) opioids administration was necessary due to the high intensity of pain. Half of the patients experienced transient rise in body temperature that was not a result of an infection. No other serious complications occurred. These observations about the safety of the procedure were in accordance with those observed previously in our department and reported in the literature [2, 27–32, 36].

In the previously published study by our team, we observed elevated values of fT4 and fT3 3 days after embolization, which showed quick reversal to near-normal or normal levels during follow-up [32]. Similarly, peak fT4 level was noted 3 days and fT3 level 7 days after embolization in present results, with a gradual normalization reached after 1 month. These observations are in agreement with these obtained by Xiao et al. [2], Zhao et al. [34, 37], and Dedecjus et al. [29]. This phenomenon may be explained by the destruction of thyroid parenchyma due to acute ischemia and necrosis of glandular tissue. Despite this transient post-procedure thyrotoxicosis, no symptoms of hyperthyroidism in euthyroid subjects appeared, as well as no symptoms worsening in patients already diagnosed as having hyperthyroidism. That may be partly attributed to prednisone and beta-adrenergic blocking agents administration.

The goiter volume reduction seems to be one of the most important aim in this kind of treatment. In previous studies, thyroid volume was assessed using CT scanning performed shortly after the therapy or based only on clinical examination [2, 29]. Physical examination, as well as ultrasonography, could not provide precise estimation of thyroid volume, especially in case of retrosternal goiter. For that reason, CT performed before and after the arterial embolization (at the end of the observation time) seems to be the most applicable imaging technique to assess and compare the thyroid volume values. Generally, the mean change in thyroid volume was highly statistically significant in the present study (51.58 ml—37 %) and the reduction in subjects with three obliterated arteries (78.8 ml—43.8 %) corresponded with the clinical observation of Xiao et al. [2] Previously, CT examination performed 3 months after embolization visualized goiter volume reduction of approximately 32 % of its original volume (from 13.0 to 76.3 %) with mean thyroid volume of 94 ml [32]. Decrease in the size of goiter was accompanied with the compression symptoms improvement, especially in patients with a huge goiter.

The authors are aware that the enrolled group of patients is non-homogenous. The main criterion of inclusion was the inability to use the standard methods of treatment. Hence, thyroid arterial embolization seems to be an alternative or so-called last chance therapy and it should be performed on a large number of different disorders to prove its true applicability.

In conclusion, thyroid arterial embolization seems to be a worth considering novel treatment modality of thyroid disorders. This study suggests that this technique is rather safe, with no serious post-procedure side effects, no persistent calcium–phosphate balance disruptions, and short time of total anesthesia needed. Thyroid arterial embolization provided normalization of thyroid function in 70.6 % of subjects and positive modulation of thyroid autoimmune processes. By the reduction of thyroid volume improvement of compression symptoms appears. However, due to small number of patients included and limited data from other medical centers, more prospective, randomized researches are needed to confirm these promising results.
